# Racial/ethnic disparity in the associations of smoking status with uncontrolled hypertension subtypes among hypertensive subjects

**DOI:** 10.1371/journal.pone.0182807

**Published:** 2017-08-09

**Authors:** Xuefeng Liu, Tinghui Zhu, Milisa Manojlovich, Hillel W. Cohen, Dennis Tsilimingras

**Affiliations:** 1 Department of Systems, Population, and Leadership, University of Michigan School of Nursing, Ann Arbor, MI, United States of America; 2 Frankel Cardiovascular Center, University of Michigan School of Medicine, Ann Arbor, MI, United States of America; 3 Department of Epidemiology and Population Health, Albert Einstein College of Medicine, Bronx, NY, United States of America; 4 Department of Family Medicine & Public Health Sciences, Wayne State University School of Medicine, Detroit, MI, United States of America; University College London, UNITED KINGDOM

## Abstract

**Background:**

Racial/ethnic differences in the associations of smoking with uncontrolled blood pressure (BP) and its subtypes (isolated uncontrolled systolic BP (SBP), uncontrolled systolic-diastolic BP, and isolated uncontrolled diastolic BP (DBP)) have not been investigated among diagnosed hypertensive subjects.

**Methods:**

A sample of 7,586 hypertensive patients aged ≥18 years were selected from the National Health and Nutrition Examination Survey 1999–2010. Race/ethnicity was classified into Hispanic, non-Hispanic white, and non-Hispanic black. Smoking was categorized as never smoking, ex-smoking, and current smoking. Uncontrolled BP was determined as SBP≥140 or DBP≥90 mm Hg. Isolated uncontrolled SBP was defined as SBP≥140 and DBP<90 mm Hg, uncontrolled SDBP as SBP≥140 and DBP≥90 mm Hg, and isolated uncontrolled DBP as SBP<140 and DBP≥90 mm Hg. Adjusted odds ratios (ORs) with 95% confidence intervals (CIs) of uncontrolled BP and its subtypes were calculated using weighted logistic regression models.

**Results:**

The interaction effect of race and smoking was significant after adjustment for the full potential confounding covariates (Adjusted p = 0.0412). Compared to never smokers, current smokers were 29% less likely to have uncontrolled BP in non-Hispanic whites (OR = 0.71, 95% CI = 0.56–0.90), although the likelihood for uncontrolled BP is the same for smokers and never smokers in Hispanics and non-Hispanic blacks. Current smokers were 26% less likely than never smokers to have isolated uncontrolled SBP in non-Hispanic whites (OR = 0.74, 95% CI = 0.58–0.95). However, current smoking is associated with an increased likelihood of uncontrolled systolic-diastolic BP in non-Hispanic blacks, and current smokers in this group were 70% more likely to have uncontrolled systolic-diastolic BP than never smokers (OR = 1.70, 95% CI = 1.10–2.65).

**Conclusion:**

The associations between current smoking and uncontrolled BP differed over race/ethnicity. Health practitioners may need to be especially vigilant with non-Hispanic black smokers with diagnosed hypertension.

## Introduction

According to the Disease Control and Centers Prevention (CDC), nearly 36 million adults have uncontrolled blood pressure (BP) in the United States (US) although most of them have health insurance and have actually seen a doctor twice a year [1]. Lack of BP control dramatically increases the risks of cardiovascular diseases among hypertensive patients [2–7]. While remarkable advances in therapy have provided the capability for lowering BP in persons with hypertension, uncontrolled BP continues to be a major public health concern with the number of people with uncontrolled BP increasing [8].

Uncontrolled BP varies across racial groups [9]. Racial difference in uncontrolled BP may be attributed to racial differences in risk factors and their associations with elevated BP. Smoking has been linked to uncontrolled BP [10–14]. However, the association of smoking with uncontrolled BP remains inconclusive, and how this association varies with race/ethnicity has not been investigated. A few studies have shown that smoking causes an acute BP elevation and is positively associated with severe uncontrolled BP [11, 12]; several other studies have observed that smokers generally have lower BP than nonsmokers [13, 14]. Racial/ethnic composition of the study population may play an important role in explaining the paradoxical associations between smoking and uncontrolled BP in different studies. The paradoxical associations might be attributed to the racial difference in the association of smoking with uncontrolled BP. Moreover, uncontrolled BP includes three subtypes: Isolated uncontrolled systolic BP (SBP), uncontrolled systolic-diastolic BP (SDBP), and isolated uncontrolled diastolic BP (DBP) [1]. These subtypes may be associated with different risk factors including race/ethnicity and smoking, and may play a different role in predicting the risk of cardiovascular disease. Examining racial/ethnic differences in the association of smoking status with uncontrolled BP, particularly with uncontrolled BP subtypes among diagnosed hypertensive patients would be critical for clarifying the confusion about inconclusive smoking-BP associations and better understanding the racial differences in uncontrolled BP and cardiovascular risk.

In this study, data of BP, smoking, race/ethnicity and confounding covariates on hypertensive subjects aged 18 years or above were collected from the continuous National Health and Nutrition Examination Survey (NHANES) 1999–2010. We hypothesize that smoking is associated with uncontrolled BP, isolated uncontrolled SBP, uncontrolled systolic-diastolic BP, and isolated uncontrolled DBP in different ways among Hispanics, non-Hispanic whites, and non-Hispanic blacks. The goal of this study was to identify racial/ethnic disparities in the relationships of smoking status and uncontrolled BP outcomes. These differences would allow health practitioners to target race-specific associations in an effort to better control BP and thereby reduce the risk of cardiovascular-related morbidities and mortality among hypertensive patients.

## Methods

### Study design and participants

The continuous NHANES program which began in 1999 is conducted in every other year by the National Center for Health Statistics in the CDC [15]. It is designed to assess the health and nutrition status of people in the US. NHANES is a biennial comprehensive survey including a series of cross-sectional nationally representative health interview and examination surveys. In each two-year cycle of the survey, approximately 10,000 participants representative of the civilian non-institutionalized US population are selected in different geographic areas across the country, using a complex stratified multistage probability cluster sampling design. Interview components are performed at the participant’s house and examination and lab components are measured in the mobile examination centers. All the participants sign an informed consent form, and the study has approval from the National Center for Health Statistics Institutional Review Board. More information can be found in measurement procedures and protocols on the NHANES website available at http://www.cdc.gov/nchs/about/major/nhanes/datalink.htm, or in the previous studies [10, 16].

We combined 6 cycles of NHANES surveys from 1999–2000 to 2009–2010 for this study. Participants aged< 18 years who were interviewed but not examined, who were not of Hispanic, non-Hispanic white, and non-Hispanic black origin, who were pregnant women (Pregnancy in women determined by a self-reported questionnaire and a urine pregnancy test), and who were not diagnosed as hypertensive patients were excluded from the study. In addition, participants with missing BP measurements and missing information for smoking status were also excluded. There were a total of 33,560 individuals aged 18 years or above who participated in both the interview and examination; out of these, 32,148 individuals were classified into one of three racial/ethnic groups as given below. After excluding 1,258 pregnant women, we had 30,890 subjects included among which 8,187 were diagnosed with hypertension. Excluding those individuals with missing values for BP and smoking, we included 7,586 individuals with diagnosed hypertension in the final sample.

### Measurements

#### Outcome variables

Each participant had up to 4 readings of SBP and DBP recorded manually by trained physicians using mercury sphygmomanometers and appropriately sized arm cuffs after 5 minutes of resting in a seated position [17]. The SBP and DBP for each individual were calculated as the averages of SBP and DBP readings with the first reading excluded. A participant was defined to have diagnosed hypertension if s/he self-reported hypertension diagnosed by a doctor which was further confirmed by elevated BP (SBP ≥140 mm Hg or DBP ≥90 mm Hg), or was currently taking prescribed medications for hypertension control at the time of examination. According to the Seventh Report of the Joint National Committee on Prevention, Detection, Evaluation and Treatment of High Blood Pressure [18], uncontrolled BP was determined as having SBP ≥140 mm Hg or DBP ≥90 mm Hg among hypertensive patients. For patients with uncontrolled BP, isolated uncontrolled SBP was defined as having SBP ≥140 mm Hg and DBP < 90 mm Hg; uncontrolled SDBP was defined as having SBP ≥140 mm Hg and DBP ≥90 mm Hg; isolated uncontrolled DBP was defined as having SBP < 140 mm Hg and DBP ≥90 mm Hg.

#### Explanatory variables

Two explanatory variables of interest included in this study were race/ethnicity and smoking status. Race/ethnicity was determined by self-report and classified into Hispanic (including Mexican American and other Hispanic), non-Hispanic white, and non-Hispanic black. Smoking information was obtained from a household interview for subjects aged ≥20 years and from the mobile examination center for subjects aged 18–19 years. Smoking status was defined by a subject’s response to several questions related to smoking history [10]. It was categorized into three groups: never smoking, ex-smoking, and current smoking. Smokers were defined as subjects aged ≥20 years who had responded “Yes” to the question “Have you smoked at least 100 cigarettes in your entire life?” or subjects aged 18 to 19 years who had positively responded to the question “Have you ever tried cigarette smoking, even 1 or 2 puffs?”. Smokers were comprised of current and ex-smokers. Current smokers included those aged ≥20 years who reported current smoking by response to the question “Do you now smoke cigarettes?” or those aged 18–19 years who reported smoking in the past 5 days by a response to the question “During the past 5 days, did you use any product containing nicotine including cigarettes, pipes, cigars, chewing tobacco, snuff, nicotine patches, nicotine gum, or any other product containing nicotine?” Ex-smokers included those aged ≥20 years who did not report current smoking but had smoked at least 100 cigarettes in their lifetime and those 18–19 years who did not report smoking in the past 5 days but had tried a cigarette in the past.

#### Clinical covariates

High cholesterol and diabetes are associated with increased risk of uncontrolled BP [19, 20], and may confound the association between smoking and uncontrolled BP. We included them as potential clinical confounders to control for the confounding errors. A participant was defined to have high cholesterol if his/her serum cholesterol ≥200 mg/dl. Serum cholesterol was measured by using the Roche/Hitachi Modular P Chemistry Analyzer (Genentech, Inc., South San Francisco, CA). Diabetes was determined by clinical measurements on glycohemoglobin levels and a positive response to any of the following questions, “Have you ever been told by a doctor that you have diabetes?”; “Are you now taking insulin?”; and “Are you now taking diabetes pills to lower your blood sugar?” [15, 21]. A participant was defined as having diabetes if s/he was taking medications for diabetes, or had been told by a doctor to have diabetes further confirmed by elevated levels of glycohemoglobin ≥6.5%. Glycohemoglobin measurements were performed on the A1c G7 HPLC Glycohemoglobin Analyzer (Tosoh Medics, Inc., South San Francisco, CA)

Urinary albumin to creatinine ratio (UACR) has been linked to hypertension [22]. In this study, we included it to adjust for the confounding impact. The levels of albuminuria were determined by a urinary albumin to creatinine ratio (UACR). A participant was defined as having microalbuminuria if his/her UACR level was ≥30 mg/g and <300 mg/g, and having macroalbuminuria if his/her UACR level was ≥300 mg/g [23]. Urinary albumin was measured in a solid-phase fluorescent immunoassay using a Sequoia-Turner fluorometer (Mountain View, CA), and urinary creatinine was measured colorimetrically by a Jaffé rate reaction on a Beckman Synchron AS/ASTRA clinical analyzer (Beckman Instruments, Brea, CA).

#### Other covariates

Information on age, gender, education, and use of medication for hypertension was self-reported. Age was categorized into three groups: 18–39 years (young adults), 40–59 years (middle-aged adults), and 60 years or above (old adults). The level of education was classified as high school or below and college or above, based on the subject’s number of years in school. The use of medication for hypertension was determined by a positive response to the question “Are you now taking prescribed medicine for HBP (high blood pressure)?”. Poverty was defined as a family’s poverty income ratio <1.0; the poverty income ratio was calculated by dividing the family total income by the appropriate poverty threshold specific to family size as well as the appropriate year and state, issued each year by the Department of Health and Human Services. A subject was determined to have generalized obesity if body mass index was ≥30 kg/m^2^.

### Statistical analysis

The NHANES supporting and analytical guidelines for surveys 1999–2010 were followed [24]. Stratum, cluster and weight design techniques for survey data were incorporated into the data analysis to ensure the representativeness and generalization of the point estimates. Means or percentages and 95% confidence intervals (CIs) were calculated to examine differences in characteristics of subjects with diagnosed hypertension over racial/ethnic groups of Hispanics, non-Hispanic whites, and non-Hispanic blacks and over smoking status. Differences in means or percentages of characteristics were tested for significance by using χ^2^ statistics for categorical variables and Wald F tests for continuous variables. The means or rates of characteristics were all age-adjusted by direct standardization to the US 2000 census population with diagnosed hypertension except for age specific estimates.

After controlling for the potential confounding factors, adjusted odds ratios (ORs) with 95% CIs of uncontrolled BP, isolated uncontrolled SBP, uncontrolled SDBP, and isolated uncontrolled DBP were obtained from weighted logistic regression models to examine the associations of current and ex-smoking with uncontrolled BP outcomes. The models used to examine the association were adjusted for age, gender, race/ethnicity, education, family poverty income ratio, body mass index, serum cholesterol, diabetes, albumin-to-creatinine ratio, and antihypertensive medication for hypertension. In logistic regression that involves multiple independent variables, conducting separate tests (each at the 0.05 level) would inflate the overall family-wise error rate for the analysis. Consequently, a Bonferroni-type adjustment were used to provide some protection for the overall error rate. In this study, instead of recalculating the significance level α, we recalculated the adjusted p values based on the original p values from the regression analysis to account for the adjustment for multiple comparison by using the step-down Bonferroni method for logistic regression. All the data analyses were performed on windows 7 PC using survey procedures in SAS version 9.4 (SAS Institute Inc, Cary, NC).

## Results

### General information of the study sample

The average age of study participants was 60.52 years. After adjustment for age, 54.15% of participants were females; 7.80% were Hispanics and 14.67% were non-Hispanic Blacks (Table 1). The rates of current smoking and ex-smoking were 21.51% and 35.07%, respectively. The overall rate of uncontrolled BP was 48.13%.

**Table 1:** Characteristics of subjects with diagnosed hypertension by race/ethnicity in NHANES 1999–2010.

Characteristics	All	Means or percentages (95% confidence intervals)		
		Hispanic	Non-Hispanic white	Non-Hispanic black
Count (N)	7,586	1,542	4,052	1,992
Age				
Mean (years)	60.52 (59.97, 61.08)	57.58 (56.09, 59.07)	61.60 (60.90, 62.30)	56.43 (55.70, 57.16) ‡
18–39 (%)	6.71 (5.82, 7.59)	9.90 (6.83, 12.97)	5.78 (4.69, 6.86)	9.89 (8.35, 11.43) ‡
40–59 (%)	39.76 (38.12, 41.39)	43.93 (38.84, 49.02)	37.48 (35.37, 39.58)	49.52 (46.86, 52.18) ‡
≥60 (%)	53.54 (51.66, 55.41)	46.17 (40.93, 51.41)	56.75 (54.37, 59.13)	40.59 (37.84, 43.34) ‡
Gender				
Female (%)	54.15 (52.67, 55.64)	55.86 (52.10, 59.62)	52.45 (50.70, 54.20)	61.29 (58.87, 63.70) ‡
Education				
High school or below (%)	52.90 (50.62, 55.18)	71.97 (68.79, 75.15)	49.40 (46.27, 52.52)	62.17 (59.07, 65.26) ‡
Family poverty income ratio				
Mean	2.93 (2.85, 3.01)	2.01 (1.85, 2.16)	3.14 (3.03, 3.24)	2.33 (2.22, 2.45) ‡
Poor (%)	11.68 (10.60, 12.75)	29.74 (23.35, 36.12)	8.16 (6.86, 9.45)	22.10 (19.22, 24.97) ‡
Body mass index				
Mean (kg/m^2^)	31.02 (30.82, 31.23)	30.73 (30.28, 31.17)	30.88 (30.62, 31.14)	31.98 (31.66, 32.31) †
Obesity (%)	49.29 (47.97, 50.61)	48.88 (44.81, 52.95)	48.39 (46.83, 49.94)	55.04 (52.72, 57.35) *
Smoking status				
Ex-smoking (%)	35.07 (33.81, 36.33)	27.09 (24.51, 29.68)	37.42 (35.76, 39.09)	27.27 (25.32, 29.22) ‡
Current smoking (%)	21.51 (20.12, 22.89)	18.77 (16.27, 21.28)	21.05 (19.32, 22.77)	26.54 (23.85, 29.23) *
Serum cholesterol				
Mean (mg/dl)	201.44 (199.93, 202.96)	203.73 (200.53, 206.93)	201.42 (199.60, 203.24)	201.00 (198.02, 203.97)
High cholesterol (%)	49.00 (47.34, 50.66)	49.58 (46.17, 52.99)	49.15 (47.10, 51.21)	48.61 (45.72, 51.50)
Diabetes (%)	24.37 (23.11, 25.64)	36.95 (32.66, 41.24)	21.04 (19.60, 22.48)	36.71 (34.55, 38.87) ‡
Albuminuria				
Microalbuminuria (%)	15.05 (14.06, 16.03)	22.38 (19.45, 25.31)	13.56 (12.44, 14.68)	18.20 (16.40, 19.99) ‡
Macroalbuminuria (%)	3.79 (3.29, 4.29)	7.01 (5.28, 8.74)	2.96 (2.39, 3.53)	6.36 (5.26, 7.46) ‡
Currently taking medications for hypertension (%)	94.22 (93.46, 94.99)	92.72 (90.80, 94.63)	94.41 (93.42, 95.39)	94.28 (93.34, 95.21)
Uncontrolled BP (%)	48.13 (46.31, 49.94)	58.05 (54.41, 61.68)	45.59 (43.42, 47.76)	55.75 (53.53, 57.96) †
Isolated uncontrolled SBP (%)	32.88 (31.49, 34.27)	36.47 (33.52, 39.41)	32.51 (30.73, 34.29)	33.40 (31.57, 35.24)
Isolated uncontrolled DBP (%)	4.85 (4.16, 5.53)	5.66 (3.83, 7.50)	4.80 (3.94, 5.67)	4.75 (3.76, 5.74)
Isolated uncontrolled SDBP (%)	10.40 (9.30, 11.50)	15.92 (13.13, 18.71)	8.28 (7.07, 9.49)	17.60 (15.96, 19.24) ‡

Notes: BP, blood pressure; DBP, diastolic blood pressure; SBP, systolic blood pressure; SDBP, systolic-diastolic blood pressure.

Data were age-adjusted by direct standardization to the US 2000 census population except for age-specific estimates.

‡ P<0.001

† P<0.01

* P<0.05, for the significance of the overall difference of means or percentages of characteristics over smoking status.

### Characteristics by race/ethnicity and smoking status

We compared characteristics with missing data on the explanatory and outcome variables (BP/smoking/ethnicity) with those with complete data in terms of other covariates to indicate whether the selected sample is biased. The comparison results are presented in S1 Table. From this Table, we can see that there were no significant differences in the covariates between missing and complete data, and the selected sample was not significantly biased by missing data on BP/smoking/ethnicity.

Compared with non-Hispanic whites, Hispanics and non-Hispanic blacks were younger, less educated, poorer, and had lower rates of ex-smoking and higher rates of diabetes (p<0.0001), albuminuria (both microalbuminuria and macroalbuminuria, p<0.0001), and uncontrolled BP (p<0.01) (Table 1). The proportion of females (p<0.0001), the average BMI (p<0.01), the rate of obesity (p<0.05), and the rate of current smoking (p<0.05) were higher in non-Hispanic blacks than in Hispanics and non-Hispanic whites. However, the proportions of hypertensive participants who were currently taking medications for hypertension control were similar among the three racial/ethnic groups.

Characteristics of the study sample by smoking status in NHANES 1999–2010 is presented in Table 2. Among diagnosed hypertensive participants in this study, current smokers were younger than never smokers and ex-smokers (p<0.0001). Compared to never smokers, current smokers included fewer females and had lower education, higher poor rates, lower obesity rates (all p<0.0001), and higher rates of macroalbuminuria (p<0.01). The proportions of Hispanics were lower in current and ex-smokers than in never smokers (p<0.05); the proportion of non-Hispanic whites was higher in ex-smokers (p<0.0001). Current smokers had a higher proportion of non-Hispanic blacks than ex-smokers (p<0.0001).

**Table 2:** Characteristics of subjects with diagnosed hypertension by smoking status in NHANES 1999–2010.

Characteristics	Means or percentages (95% confidence intervals)		
	Never smoking	Ex-smoking	Current smoking
Count (N)	3,322	2,688	1,576
Age			
Mean (years)	61.15 (60.48, 61.83)	63.89 (63.17, 64.60)	53.86 (52.99, 54.72) ‡
18–39 (%)	6.26 (5.06, 7.46)	3.85 (2.66, 5.04)	12.18 (10.06, 14.30) ‡
40–59 (%)	39.46 (37.16, 41.75)	30.85 (28.12, 33.58)	54.66 (51.71, 57.61) ‡
≥60 (%)	54.28 (51.96, 56.61)	65.30 (62.44, 68.16)	33.16 (3015, 36.17) ‡
Gender			
Female (%)	68.35 (66.25, 70.45)	43.79 (40.97, 46.61)	42.70 (39.20, 46.21) ‡
Race/Ethnicity			
Hispanic (%)	9.72 (7.29, 12.15)	6.15 (4.62, 7.67)	6.86 (4.90, 8.82) *
Non-Hispanic white (%)	74.27 (70.64, 77.90)	83.34 (80.93, 85.75)	74.53 (70.45, 78.61) ‡
Non-Hispanic black (%)	16.01 (13.35, 18.67)	10.51 (8.77, 12.25)	18.61 (15.11, 22.11) ‡
Education			
High school or below (%)	50.87 (48.21, 53.52)	47.11 (44.06, 50.15)	64.73 (61.04, 68.41) ‡
Family poverty income ratio			
Mean	2.96 (2.87, 3.05)	3.16 (3.06, 3.27)	2.55 (2.44, 2.66) ‡
Poor (%)	10.21 (8.89, 11.54)	9.02 (7.68, 10.35)	19.05 (16.76, 21.34) ‡
Body mass index			
Mean (kg/m^2^)	31.55 (31.23, 31.86)	31.48 (31.02, 31.94)	29.63 (29.24, 30.01) ‡
Obesity (%)	51.76 (49.74, 53.77)	51.49 (48.74, 54.25)	41.97 (39.16, 44.78) ‡
Serum cholesterol			
Mean (mg/dl)	204.27 (202.29, 206.26)	199.24 (196.35, 202.12)	200.13 (196.74, 203.51)
High cholesterol (%)	51.79 (49.56, 54.03)	46.87 (43.50, 50.23)	47.04 (43.55, 50.54)
Diabetes (%)	23.69 (21.78, 25.61)	25.11 (22.89, 27.33)	24.56 (22.57, 26.54)
Albuminuria			
Microalbuminuria (%)	14.52 (13.04, 16.00)	15.10 (13.47, 16.73)	15.36 (13.12, 17.61)
Macroalbuminuria (%)	3.25 (2.60, 3.90)	3.44 (2.53, 4.35)	5.48 (4.11, 6.85) †
Currently taking medications for hypertension (%)	94.63 (93.51, 95.76)	94.70 (93.12, 96.28)	92.91 (91.28, 94.54)
Uncontrolled BP (%)	49.30 (46.94, 51.67)	46.86 (43.66, 50.06)	46.38 (42.93, 49.84)
Isolated uncontrolled SBP (%)	34.53 (32.60, 36.47)	31.28 (28.76, 33.80)	30.47 (27.33, 33.62)
Isolated uncontrolled DBP (%)	4.77 (3.71, 5.82)	5.43 (3.95, 6.92)	4.73 (3.61, 5.84)
Isolated uncontrolled SDBP (%)	10.01 (8.53, 11.49)	10.15 (8.36, 11.94)	11.18 (9.37, 13.00)

Notes: BP, blood pressure; DBP, diastolic blood pressure; SBP, systolic blood pressure; SDBP, systolic-diastolic blood pressure.

Data were age-adjusted by direct standardization to the US 2000 census population except for age-specific estimates.

‡ P<0.001

† P<0.01

* P<0.05 for the significance of the overall difference of means or percentages of characteristics over smoking status.

### Rates of uncontrolled BP and its subtypes by smoking status and race/ethnicity

We present rates of uncontrolled BP and its subtypes by smoking status within each racial/ethnic group in Table 3. Corresponding statistical tests were conducted for bi-variate association between smoking and uncontrolled BP. Compared to never smokers, current smokers have a higher rate of uncontrolled SDBP in the non-Hispanic black population. The difference in rates of uncontrolled BP subtypes between smokers and never smokers were not significant in other racial/ethnic groups.

**Table 3:** Percentages of uncontrolled blood pressure and subtypes by smoking status within each racial/ethnic group.

Characteristics	Count	Percentages (95% confidence intervals)		
		Never smoking	Ex-smoking	Current smoking
Hispanic				
Uncontrolled BP (%)	895	58.56 (53.65, 63.46)	55.68 (47.40, 63.95)	56.75 (47.91, 65.59)
Isolated uncontrolled SBP (%)	638	37.34 (33.31, 41.36)	32.11 (25.62, 38.60)	38.03 (28.94, 47.11)
Isolated uncontrolled DBP (%)	57	5.07 (1.78, 8.37)	7.69 (2.52, 12.86)	5.60 (2.25, 8.94)
Uncontrolled SDBP (%)	200	16.15 (12.51, 19.78)	15.88 (11.17, 20.58)	13.12 (8.28, 17.96)
Non-Hispanic white				
Uncontrolled BP (%)	1,922	46.50 (43.36, 49.65)	45.55 (41.95, 49.15)	42.28 (37.97, 46.59)
Isolated uncontrolled SBP (%)	1,490	34.26 (31.73, 36.79)	30.81 (27.88, 33.74)	29.80 (25.87, 33.74)
Isolated uncontrolled DBP (%)	132	4.60 (3.27, 5.94)	5.47 (3.76, 7.18)	4.55 (3.13, 5.97)
Uncontrolled SDBP (%)	300	7.64 (5.84, 9.44)	9.27 (7.32, 11.22)	7.93 (5.87, 9.98)
Non-Hispanic black				
Uncontrolled BP (%)	1,111	56.07 (52.93, 59.20)	52.22 (47.16, 57.28)	58.29 (53.49, 63.10)
Isolated uncontrolled SBP (%)	662	35.30 (32.20, 38.39)	34.65 (30.32, 38.98)	30.32 (25.86, 34.77)
Isolated uncontrolled DBP (%)	92	5.07 (3.82, 6.33)	3.13 (1.25, 5.01)	5.05 (3.41, 6.70)
Uncontrolled SDBP (%)	357	15.70 (13.24, 18.15)	14.44 (10.92, 17.96)	22.92 (19.20, 26.65) *

Notes: BP, blood pressure; DBP, diastolic blood pressure; SBP, systolic blood pressure; SDBP, systolic-diastolic blood pressure.

Data were age-adjusted by direct standardization to the US 2000 census population except for age-specific estimates.

* P<0.05 for the significance of uncontrolled BP and its subtypes across smoking categories in the three racial groups.

BP, blood pressure; DBP, diastolic blood pressure; SBP, systolic blood pressure; SDBP, systolic-diastolic blood pressure.

### Associations of smoking status with uncontrolled BP and its subtypes by race/ethnicity

We conducted logistic regression by first including the interaction of race and smoking in the full model and the adjusted p value for the interaction effect was estimated as 0.0412. In light of statistically significant interaction, we proceeded with the stratified analysis of the associations between smoking and uncontrolled BP by race/ethnicity. Adjusted ORs and 95% CIs of uncontrolled BP and its subtypes (including isolated uncontrolled SBP, uncontrolled SDBP, and isolated uncontrolled DBP) associated with ex- and current smoking are listed in Table 4. After adjustment for the full potential confounding factors, current smokers were 29% less likely to have uncontrolled BP (OR = 0.71, 95% CI = 0.56–0.90) than never smokers in non-Hispanic whites. There were no significant associations between smoking and uncontrolled BP in Hispanics and non-Hispanic blacks.

**Table 4:** Adjusted odds ratios of uncontrolled blood pressure subtypes associated with ex-smoking and current smoking (vs. never smoking) by race among diagnosed hypertensive subjects in NHANES 1999–2010.

Models for BP outcomes	Odds ratios (95% confidence intervals)		
	Hispanic	Non-Hispanic white	Non-Hispanic black
Uncontrolled BP			
Ex-smoking	0.75 (0.46, 1.21)	0.96 (0.79, 1.18)	0.81 (0.64, 1.04)
Current smoking	0.85 (0.50, 1.46)	0.71 (0.56, 0.90) †	1.08 (0.81, 1.44)
Isolated uncontrolled SBP			
Ex-smoking	0.98 (0.63, 1.54)	0.97 (0.79, 1.19)	0.88 (0.66, 1.19)
Current smoking	1.16 (0.61, 2.23)	0.74 (0.58, 0.95) *	0.82 (0.58, 1.18)
Uncontrolled SDBP			
Ex-smoking	0.53 (0.25, 1.14)	1.10 (0.71, 1.72)	0.86 (0.55, 1.35)
Current smoking	0.59 (0.27, 1.26)	0.63 (0.35, 1.14)	1.70 (1.10, 2.65) *
Isolated uncontrolled DBP			
Ex-smoking	0.41 (0.11, 1.55)	1.15 (0.65, 2.05)	1.03 (0.54, 1.98)
Current smoking	0.70 (0.23, 2.13)	0.79 (0.43, 1.46)	0.57 (0.18, 1.74)

Notes: BP, blood pressure; DBP, diastolic blood pressure; SBP, systolic blood pressure; SDBP, systolic-diastolic blood pressure.

Data were adjusted for age, gender, education, family poverty income ratio, body mass index, serum cholesterol, diabetes, albumin-to-creatinine ratio, and antihypertensive medication for hypertension.

† Adjusted P<0.01

* Adjusted P<0.05 for the significance of odds ratios of uncontrolled BP and its subtypes associated with smoking status.

Current smokers were 26% less likely to have isolated uncontrolled SBP than never smokers (OR = 0.74, 95% CI = 0.58–0.95) in non-Hispanic whites. However, current smoking was associated with an increased likelihood of uncontrolled SDBP in non-Hispanic blacks, and current smokers in this group were 70% more likely to have uncontrolled SDBP than never smokers (OR = 1.70, 95% CI = 1.10–2.65).

## Discussion

This study examined racial differences in the association of smoking status with uncontrolled BP, isolated uncontrolled SBP, uncontrolled SDBP, and isolated uncontrolled DBP among diagnosed hypertensive patients in NHANES 1999–2010. One finding that stands out was the positive association of smoking and uncontrolled SDBP in non-Hispanic blacks. Compared to never smokers, current smokers were more likely to have uncontrolled SDBP in this racial/ethnic group. African Americans metabolize nicotine more slowly and inhale higher doses of nicotine per cigarette than white smokers [25]. The average level of serum cotinine (a proximate metabolite and marker of nicotine) has been shown to be higher in African American smokers than in whites smoking the same number of cigarettes per day [26]. Higher levels of nicotine and cotinine is related to enhanced activation of the renin-angiotensin-aldosterone system and increased expression levels of angiotensin II that may partly contribute to the greater vasoconstriction and raised SBP and DBP in black smokers [27].

Current smokers were shown to be less likely to have uncontrolled BP and isolated uncontrolled SBP than never smokers in non-Hispanic whites. The associations were significant only in the model with adjustment for the full potential confounders, and could be influenced by different combinations of confounding factors, such as age, gender, race/ethnicity, education, and family poverty income ratio, etc. This indicates that smoking may play an important and independent role in uncontrolled BP and SBP in non-Hispanic whites while the associations were not robust as those between medication use and uncontrolled BP outcomes. The negative association in white patients is consistent with the findings from previous studies that targets the general population consisting of different racial/ethnic groups [12, 28, 29]. Our supportive results in non-Hispanic whites shows that the negative relationship between current smoking and uncontrolled BP found in previous studies might be dominated by non-Hispanic whites as non-Hispanic whites account for the majority of the US population. This paradoxical association may be attributed to two potential pathways. One pathway is that smokers may increase awareness of their health status so that they do more physical activity, reduce sodium intake, and reduce alcohol use to improve their health behaviors and lower their BP [11, 30]. The other pathway is that smoking is associated with appetite suppression and weight loss, and weight control is associated with lower risk of uncontrolled BP [30, 31]. This is supported by the result from the current study that average body mass index and obesity rate were lower in current smokers than in never smokers.

The associations of current smoking with uncontrolled BP varied among Hispanic, non-Hispanic whites, and non-Hispanic blacks. The interactions of smoking with other factors (e.g. age, gender, and obesity) may differ over race/ethnicity, and this difference may change the magnitude and direction of the association between smoking and SBP or DBP levels [11] and thus uncontrolled BP in hypertensive subjects in these racial/ethnic groups. Racial differences in nicotine metabolism may also play a role in this regard [32]. The analysis of uncontrolled BP subtypes showed that current smokers were less likely to have isolated uncontrolled SBP in non-Hispanic whites, but more likely to have uncontrolled SDBP in non-Hispanic blacks after adjustment for the potential confounding factors. The directionally different associations of current smoking with isolated uncontrolled SBP in non-Hispanic whites and uncontrolled SDBP in non-Hispanic blacks may finally determine the direction of the relationship between current smoking and uncontrolled BP in these two racial/ethnic groups.

There were several limitations in this study. Continuous NHANES was a series of national surveys with cross sectional design. The results could not be used for causal effects of smoking on the risk of uncontrolled hypertension in different racial populations. The NHANES survey did not provide a detailed list of specific medications that were used for hypertensive control. Even though we controlled for the confounding impact of taking medications for hypertension, we could not obtain information regarding the adherence to medication, the treatment duration, and the specific dose of medication use. These factors might influence the results in the present study [33, 34]. Other factors we could not consider due to the sample and design limitations, such as diet, sodium intake and physical activity, may confound the association of smoking with uncontrolled BP. While some specific groups in the NHANES were oversampled and unequal probabilities of selection were taken into account, lack of power due to small cell counts—especially of rare outcome events (e.g. isolated uncontrolled DBP in Hispanics which matter most in logistic regression) and wide confidence intervals may raise strong limitations regarding the potential of both Type I and Type II error and even the direction of the associations observed. Finally, diagnosed hypertension in this study was determined in terms of self-reported hypertension diagnosed by a health professional, average SBP and DBP measurements, and the use of prescribed antihypertensive medications for each individual. Our definition of hypertension excluded the hypertensive persons with BP successfully controlled by physical activity, weight control and other non-pharmacological techniques.

## Perspective

The associations between current smoking and uncontrolled BP varied with race/ethnicity among diagnosed hypertensive subjects. Current smokers were more likely than never smokers to have uncontrolled SDBP in non-Hispanic blacks. In this regard, health practitioners need to be especially vigilant with non-Hispanic black hypertensive subjects who are currently smoking to improve control of both high SBP and high DBP in this racial group. Even though current smokers were less likely to have uncontrolled BP and isolated uncontrolled SBP in non-Hispanic whites, the negative association should not indicate that smoking would have beneficial effects on the uncontrolled BP or isolated uncontrolled SBP in this group, considering cigarette smoking is the leading preventable cause of diseases and deaths in the US [35, 36], irrespective of race/ethnicity. The mechanism for the controversial associations between current smoking and uncontrolled BP subtypes among hypertensive subjects in non-Hispanic whites and blacks remains unknown and needs to be further investigated.

## Supporting information

S1 Table Comparison of missing data on the explanatory and outcome variables (BP/smoking/ethnicity) with those with complete data in terms of other covariates. (DOCX)

## References

[1] Centers for Disease Control and Prevention. Vital Signs: Awareness and Treatment of Uncontrolled Hypertension Among Adults—United States, 2003–2010. MMWR 2012; 61:703–709. 22951452

[2] National Lung, Heart, Blood Institute. Effect of High Blood Pressure on Your Body. Available at http://www.nhlbi.nih.gov/hbp/hbp/effect/effect.htm. Accessed by September 14, 2013.

[3] Hyman DJ, Pavlik VN. Uncontrolled hypertension as a risk for coronary artery disease: Patient characteristics and the role of physician intervention. Current Atherosclerosis Reports 2003; 5:132–138. 10.1007/s11883-003-0085-z 12573199

[4] Klungel OH, Kaplan RC, Heckbert SR, Smith NL, Lemaitre RN, Longstreth WT Jr et al Control of blood pressure and risk of stroke among pharmacologically treated hypertensive patients. Stroke 2000; 31(2):420–424. 10657416 10.1161/01.str.31.2.420

[5] Iyer AS, Ahmed MI, Filippatos GS, Ekundayo OJ, Aban IB, Love TE et al Uncontrolled hypertension and increased risk for incident heart failure in older adults with hypertension: findings from a propensity-matched prospective population study. J Am Soc Hypertens 2010; 4:22–31. doi: 10.1016/j.jash.2010.02.002 20374948 10.1016/j.jash.2010.02.002 PMC2914566

[6] Lloyd-Jones DM, Evans JC, Levy D. Hypertension in Adults across the Age Spectrum: Current Outcomes and Control in the Community. JAMA 2005; 294:466–472. doi: 10.1001/jama.294.4.466 16046653 10.1001/jama.294.4.466

[7] American Heart Association. High blood pressure. Available at http://www.heart.org/HEARTORG/Conditions/HighBloodPressure/High-Blood-Pressure_UCM_002020_SubHomePage.jsp. Accessed by October 2, 2016.

[8] Kearney PM, Whelton M, Reynolds K, Muntner P, Whelton PK, He J. Global burden of hypertension: analysis of world-wide data. Lancet 2005; 365:217–223. 10.1016/S0140-6736(05)17741-1 15652604

[9] Egan BM, Zhao Y, Axon RN. US trends in prevalence, awareness, treatment, and control of hypertension, 1988–2008. JAMA 2010; 303:2043–2050. doi: 10.1001/jama.2010.650 20501926 10.1001/jama.2010.650

[10] Liu XF, Byrd JB. Cigarette Smoking and Subtypes of Uncontrolled Blood Pressure Among Diagnosed Hypertensive Patients: Paradoxical Associations and Implications. American Journal of Hypertension 2017; 30(6):602–609. doi: 10.1093/ajh/hpx014 28203691 10.1093/ajh/hpx014

[11] Primatesta P, Falaschetti E, Gupta S, Marmot MG, Poulter NR. Association between smoking and blood pressure: evidence from the Health Survey for England. Hypertension 2001; 37:187–193. 11230269 10.1161/01.hyp.37.2.187

[12] McNagny SE, Ahluwalia JS, Clark WS, Resnicow KA. Cigarette smoking and severe uncontrolled hypertension in inner-city African Americans. Am J Med 1997; 103:121–127. 9274895 10.1016/s0002-9343(97)00131-9

[13] Mikkelsen KL, Wiinberg N, Høegholm A, Christensen HR, Bang LE, Nielsen PE et al Smoking related to 24-h ambulatory blood pressure and heart rate: a study in 352 normotensive Danish subjects. Am J Hypertens 1997; 10:483–491. 9160757 10.1016/s0895-7061(96)00487-6

[14] Green MS, Jucha E, Luz Y. Blood pressure in smokers and nonsmokers: epidemiologic findings. Am Heart J. 1986; 111:932–940. 3706114 10.1016/0002-8703(86)90645-9

[15] National Center for Health Statistics; the Centers for Disease Control and Prevention (CDC). The National Health and Nutrition Examination Survey (NHANES) 1999–2010 Data Files. Available at: http://www.cdc.gov/nchs/nhanes/nhanes_questionnaires.htm. Accessed February 26, 2013.

[16] Liu XF, Rodriguez CJ, Wang KS. Prevalence and Trends of Isolated Systolic Hypertension among Untreated Adults in the United States. Journal of the American Society of Hypertension 2015; 9(3):197–205. doi: 10.1016/j.jash.2015.01.002 25795550 10.1016/j.jash.2015.01.002 PMC4573631

[17] National Health and Nutrition Examination Survey. Physician examination procedures manual (January 2007). Available at http://www.cdc.gov/nchs/data/nhanes/nhanes_09_10/physician.pdf. Accessed February 10, 2013.

[18] Chobanian AV, Bakris GL, Black HR, Cushman WC, Green LA, Izzo JL et al Seventh report of the joint national committee on prevention, detection, evaluation, and treatment of high blood pressure. Hypertension 2003; 42:1206–1252. doi: 10.1161/01.HYP.0000107251.49515.c2 14656957 10.1161/01.HYP.0000107251.49515.c2

[19] Egan BM, Li J, Qanungo S, Wolfman TE. Blood pressure and cholesterol control in hypertensive hypercholesterolemic patients: NHANES 1988–2010. Circulation 2013; 128:29–41. doi: 10.1161/CIRCULATIONAHA.112.000500 23817481 10.1161/CIRCULATIONAHA.112.000500 PMC4066305

[20] Liu XF, Song P. Is the association of diabetes with uncontrolled blood pressure stronger in Mexican Americans and blacks than in whites among diagnosed hypertensive patients? American Journal of Hypertension 2013; 26 (11):1328–1334. doi: 10.1093/ajh/hpt109 23864584 10.1093/ajh/hpt109 PMC7500068

[21] Ong KL, Cheung BM, Wong LY, Wat NM, Tan KC, Lam KS. Prevalence, treatment, and control of diagnosed diabetes in the US. National health and nutrition examination survey 1999–2004. Annals of epidemiology 2008; 18:222–229. doi: 10.1016/j.annepidem.2007.10.007 18201902 10.1016/j.annepidem.2007.10.007

[22] Sung KC, Ryu S, Lee JY, Lee SH, Cheong E, Hyun YY et al Urine Albumin/Creatinine Ratio Below 30 mg/g is a Predictor of Incident Hypertension and Cardiovascular Mortality. Journal of the American Heart Association 2016; 5:e003245 doi: 10.1161/JAHA.116.003245 27625343 10.1161/JAHA.116.003245 PMC5079007

[23] Eknoyan G, Hostetter T, Bakris GL, Hebert L, Levey AS, Parving HH et al Proteinuria and other markers of chronic kidney disease: A position statement of the national kidney foundation (nkf) and the national institute of diabetes and digestive and kidney diseases (niddk). Am J Kidney Dis. 2003; 42: 617–622. 14520612 10.1016/s0272-6386(03)00826-6

[24] Johnson CL, Paulose-Ram R, Ogden CL, Carroll MD, Kruszan-Moran D, Dohrmann SM et al National Health and Nutrition Examination Survey: Analytic guidelines, 1999–2010. National Center for Health Statistics. *Vital Health Stat*; 2(161). 2013. 25090154

[25] Pérez-Stable EJ, Herrera B, Jacob PIII, Benowitz NL. Nicotine metabolism and intake in black and white smokers. JAMA 1998; 280:152–156. 9669788 10.1001/jama.280.2.152

[26] Caraballo RS, Pechacek TF, Richter P. Racial and ethnic differences in serum cotinine levels of cigarette smokers: The third national health and nutrition examination survey, 1988–1991. JAMA 1998; 280:135–139. 9669785 10.1001/jama.280.2.135

[27] Laustiola KE, Lassila R, Nurmi AK. Enhanced activation of the renin-angiotensin-aldosterone system in chronic cigarette smokers: A study of monozygotic twin pairs discordant for smoking. Clinical Pharmacology and Therapeutics 1988; 44:426–430. 3048842 10.1038/clpt.1988.175

[28] Ong KL, Tso AW, Lam KS, Cheung BM. Gender difference in blood pressure control and cardiovascular risk factors in Americans with diagnosed hypertension. Hypertension. 2008; 51(4):1142–1148. doi: 10.1161/HYPERTENSIONAHA.107.105205 18259031 10.1161/HYPERTENSIONAHA.107.105205

[29] Douglas JG, Bakris GL, Epstein M, Ferdinand KC, Ferrario C, Flack JM et al; Hypertension in African Americans Working Group of the International Society on Hypertension in blacks. Management of high blood pressure in African Americans: consensus statement of the Hypertension in African Americans Working Group of the International Society on Hypertension in blacks. Arch Intern Med. 2003; 163(5): 525–541. 12622600 10.1001/archinte.163.5.525

[30] Lee DH, Ha MH, Kim JR, Jacobs DR Jr. Effects of smoking cessation on changes in blood pressure and incidence of hypertension: a 4-year-up study. Hypertension 2001; 37(2):194–198. 11230270 10.1161/01.hyp.37.2.194

[31] Gerhard GT, Malinow MR, DeLoughery TG, Evans AJ, Sexton G, Connor SL et al Higher total homocysteine concentrations and lower folate concentrations in premenopausal black women than in premenopausal white women. American Journal of Clinical Nutrition 1999; 70(2):252–260. 10426703 10.1093/ajcn.70.2.252

[32] Pérez-Stable EJ, Benowitz NL. Do biological differences help explain tobacco-related disparities? American Journal of Health Promotion 2011; 25 (suppl.): S8–S10. 21510792 10.4278/ajhp.25.5.c2

[33] Hicks LS, Fairchild DG, Horng MS, Orav EJ, Bates DW, Ayanian JZ. Determinants of JNC VI Guideline Adherence, Intensity of Drug Therapy, and Blood Pressure Control by Race and Ethnicity. Hypertension 2004; 44:429–434. doi: 10.1161/01.HYP.0000141439.34834.84 15326088 10.1161/01.HYP.0000141439.34834.84

[34] Hicks LS, Shaykevich S, Bates DW, Ayanian JZ. Determinants of racial/ethnic differences in blood pressure management among hypertensive patients. BMC Cardiovascular Disorders 2005; 5:16 doi: 10.1186/1471-2261-5-16 15972095 10.1186/1471-2261-5-16 PMC1173097

[35] Holford TR, Meza R, Warner KE, Meernik C, Jeon J, Moolgavkar SH et al Tobacco control and the reduction in smoking-related premature deaths in the United States, 1964–2012. JAMA 2014; 311:164–171. doi: 10.1001/jama.2013.285112 24399555 10.1001/jama.2013.285112 PMC4056770

[36] US Department of Health and Human Services. The health consequences of smoking—50 years of progress: a report of the Surgeon General. Atlanta, GA: US Department of Health and Human Services, CDC; 2014. Available at http://www.surgeongeneral.gov/initiatives/tobacco.

