# Supplementary material for: Racial/ethnic disparity in the associations of smoking status with uncontrolled hypertension subtypes among hypertensive subjects
Source: PLoS One. 2017 Aug 9;12(8):e0182807. doi: 10.1371/journal.pone.0182807 (PMC5549965; doi:10.1371/journal.pone.0182807)
Supplement: S1 Table — (DOCX) [file pone.0182807.s001.docx]

**S1 Table. Comparison of missing data on the explanatory and outcome variables (BP/smoking/ethnicity) with those with complete data in terms of other covariates**

| Characteristics | Means or percentages (95% confidence Intervals) | |
| --- | --- | --- |
|  | Complete data on BP/smoking/ethnicity | Missing data on BP/smoking/ethnicity |
| Count (N) | 7,586 | 601 |
| Age  Mean (years)  18-39 (%)  40-59 (%)  ≥60 (%) | 60.52 (59.97, 61.08)  6.71 (5.82, 7.59)  39.76 (38.12, 41.39)  53.54 (51.66, 55.41) | 60.82 (59.43, 62.21)  7.75 (4.87, 10.63)  42.97 (37.76, 48.19)  49.27 (44.40, 54.14) |
| Gender  Female (%) | 54.15 (52.67, 55.64) | 55.93 (50.61, 61.25) |
| Education  High school or below (%) | 52.90 (50.62, 55.18) | 55.27 (49.81, 60.72) |
| Family poverty income ratio  Mean  Poor (%) | 2.93 (2.85, 3.01)  11.68 (10.60, 12.75) | 2.63 (2.46, 2.82)  12.52 (8.40, 16.65) |
| Body mass index  Mean (kg/m^2^)  Obesity (%) | 31.02 (30.82, 31.23)  49.29 (47.97, 50.61) | - 1. (31.79, 33.81)   52.46 (47.68, 57.23) |
| Serum cholesterol  Mean (mg/dl)  High cholesterol (%) | 201.44 (199.93, 202.96)  49.00 (47.34, 50.66) | 198.22 (194.23, 202.20)  45.87 (39.77, 51.97) |
| Diabetes (%) | 24.37 (23.11, 25.64) | 26.53 (21.69, 31.36) |
| Albuminuria  Microalbuminuria (%) Macroalbuminuria (%) | 15.05 (14.06, 16.03)  3.79 (3.29, 4.29) | 15.79 (12.46, 19.13)  6.86 (3.99, 9.73) |
| Currently taking medications for hypertension (%) | 94.22 (93.46, 94.99) | 95.53 (94.01, 97.05) |

Notes: BP, blood pressure.

Data were age-adjusted by direct standardization to the US 2000 census population except for age-specific estimates.
